# Evaluation of the Effectiveness of a Group Intervention Approach for Nurses Exposed to Violent Speech or Violence Caused by Patients: A Randomized Controlled Trial

**DOI:** 10.5402/2011/325614

**Published:** 2011-06-18

**Authors:** Makoto Inoue, Fumiko Kaneko, Hitoshi Okamura

**Affiliations:** ^1^Graduate School of Health Sciences, Hiroshima University, 1-2-3 Kasumi, Minami-ku, Hiroshima 734-8551, Japan; ^2^Department of Nursing, Faculty of Health and Welfare, Prefectural University of Hiroshima, Mihara 723-0053, Japan

## Abstract

The purpose of this study was to evaluate the effectiveness of a group intervention approach aimed at improving the mental health of psychiatric nurses exposed to violent speech/violence. Sixty-two nurses having experienced serious episodes of violent speech/violence were enrolled in this study. A group intervention approach was used in the intervention group. For both the intervention and the control groups, evaluations were conducted at three time points. Evaluations were conducted using the Impact of Event Scale-Revised (IES-R) and Profile of Mood States (POMS). The results showed that changes in the flashback, hyper-arousal, avoidance behavior, and total scores on the IES-R and anxiety and depression scores on the POMS differed significantly between the two groups. These results suggest that a group intervention approach can lessen the psychological burden of nurses exposed to violence and reduce their mental stress.

## 1. Introduction

Nurses working in clinical practice are often exposed to violent speech and/or even actual violence [1–3]. One background factor for violent speech/violence caused by patients and affecting nurses is the fact that patients staying in hospitals (environments that are totally different from their conventional lifestyles) are likely to become irritated if their freedom is restricted or if their disease does not subside or resolve [4]. Patients often view nurses as being “persons who are willing to listen to any request” or “persons who will agree to any desire.” Under such circumstances, the above-mentioned emotions experienced by patients occasionally assume the form of violent speech/violence directed against nurses [5].

A previous study demonstrated that the risk of exposure to violent speech/violence in the workplace is higher for nurses than for any other healthcare profession [6, 7]. The frequency of exposure to aggressive speech/behavior by hospitalized patients is especially high for nurses working in psychiatric facilities, with an exposure frequency that is twice as high as that for nurses working in other specialties [8]. When exposed to violent speech/violence, nurses often endure the abuse or violence without resistance, considering it “part of their job” [9]. The tendency of nurses to have such views leads to inadequate reports of this type of event, thereby hampering the development of effective countermeasures against violent speech/violence. When exposed to violent speech/violence, psychiatric nurses tend to avoid attracting the close attention of surrounding people to the hazards to which they have been exposed, and investigators have pointed out the need to investigate the impact of this type of event on the psychological features of psychiatric nurses exposed to violence and to devise valid means of dealing with such impacts appropriately [10]. At present, however, a system for the psychological followup of nurses exposed to violent speech/violence is lacking at many medical facilities.

In our previous study [11], 141 (62.7%) of the 225 psychiatric nurses who were surveyed responded that they “have been subjected to memorable violent speech/violence,” and a diagnosis of posttraumatic stress disorders (PTSD) seemed possible in 21.3% of these nurses. Previous reports on interventions for such nurses include discussions of the possibility of securing the safety of patients and nurses by learning defensive techniques based on methods of protecting oneself using self-defense [12]. Another report focused on reducing the risk of violence by providing a comprehensive violence prevention program [13]. However, these analyses and evaluations involved many ambiguities and were primarily concerned with how to deal with violence or what actions are needed to prevent violent speech/violence. 

Under these circumstances, we attempted a group intervention approach aiming at reducing the psychological stress of psychiatric nurses exposed to violent speech/violence and analyzed changes in the psychological impacts of violent speech/violence following this intervention.

## 2. Method

### 2.1. Subjects

Of the nurses working at five psychiatric hospitals in the Chugoku and Kyushu districts of Japan with 200–300 beds/hospital, those satisfying all the following requirements were enrolled in this study:

1. experience of serious violent speech/violence,
2. a 6-month or longer career working as a psychiatric nurse at the time of the invitation to participate in this study,
3. ability to participate in all the intervention programs,
4. provision of informed consent to participate in the study,
5. not in an administrative position (director of nursing department, vice director of nursing department, or chief nurse).

### 2.2. Definition of Violent Speech/Violence

Prior to the intervention, a definition of “violent speech/violence” by patients directed at nurses was needed. Taking into account the results of our preceding questionnaire survey of what forms of violent speech/violence were seen at psychiatric facilities (a survey involving 282 subjects) and referring to published articles and the definitions prepared by the International Council of Nurses, we defined violence from three aspects: physical violence, sexual violence, and verbal abuse. A portion of verbal abuse was viewed as violent speech. Experiencing any of these types of violence from patients was rated as “exposed to violent speech/violence.” Thus, the definition of “violent speech/violence” includes

1. physically violent behaviors involving physical contact (beating, kicking, biting, or scratching) and behaviors involving physical contact using pencils, eating utensils, or other objects as weapons for stabbing,
2. sexually violent behaviors, such as touching the nurse's body, inappropriate hugging, genital display, indecent speech, and requests for sexual relations (such behaviors were counted regardless of whether they were made by male or female patients),
3. verbal abuse involving hissing, using an angry tone, or making direct or indirect threats, such as “Die,” “You're ugly,” “You're bald,” “You silly ass,” “I'll beat you,” “I'll kill you,” or “I'll remember this” (these types of expressions are hereinafter collectively called “violent speech”) and behaviors not involving physical contact such as throwing an object at the nurse, spraying water on the nurse, spitting at the nurse and kicking/damaging/destroying the door.

### 2.3. Procedure

The subjects of this study were recruited by holding orientation meetings at wards for acute psychiatric care and chronic psychiatric care, with the permission of the nursing director of each facility. The orientation meetings provided detailed information about the planned study to the nurses.

The nurses who provided informed written consent at each facility (*n* = 62) were randomly allocated using the dice method to an intervention group (*n* = 30) or a control group (*n* = 32). Then, a group approach was used for the intervention group. The author, having worked as a psychiatric nurse for 11 years, served as the moderator. Each group was composed of 3-4 members who remained in the same group for all the sessions. At the end of each session, an outline of the discussion held during that session was reported to the nursing director (or vice director) of the hospital to communicate the need for systematic actions dealing with violent speech/violence.

For both the intervention and the control groups, evaluations were conducted at three time points, that is, at baseline (immediately before the start of intervention), immediately after the 6-week intervention, and 3 months thereafter. The evaluation at 3 months after the intervention was performed for the following reasons: many previous studies conducted evaluations at 3–6 months after the end of the intervention period based on the view that the influence of a group intervention approach on psychological features persists for 3–6 months after the completion of the intervention [14, 15]; an evaluation at this time point was possible in the present study.

### 2.4. Intervention Program

When conducting the intervention, we paid attention to group psychotherapy, which can be implemented by nurses and which has been reported to be a highly efficient means of treatment [16–19]. We thus adopted a group intervention approach previously applied to patients with psychiatric diseases and their family members [20–22] and used in our previous group work trials. The program was composed of a psychotherapy-based discussion, including topics regarding means of coping with violent speech/violence or psychological impacts and stress management, as well as behavioral therapy (progressive muscle relaxation + image therapy) for a total of four sessions (once weekly for 4 weeks, 90 minutes/session).

### 2.5. Measures

Based on our previous study results [11], we selected the following variables to evaluate psychological impact and stress.

#### 2.5.1. Social and Demographic Variables

Information was collected regarding age, sex, length of nursing experience, length of work in a psychiatry department, number of persons in household, presence/absence of a spouse, presence/absence of social support, degree of satisfaction with social support, presence/absence of major episodes of violent speech/violence, and interval between the time of exposure to the violent speech/violence and the present, if any. Regarding social support, the following parameters were rated using a 4-point Likert scale: number of persons who provided social support (none to very numerous), degree of satisfaction with family support (not satisfied at all to quite satisfied), and degree of satisfaction with support from acquaintances (not satisfied at all to quite satisfied).

#### 2.5.2. Impact of Event Scale-Revised (IES-R)

The IES-R is a self-rated scale composed of 22 items designed to evaluate the effect of psychological trauma. The scale was devised by Weiss and Marmer [23] as a revised version of the Impact of Event Scale created by Horowitz et al. [24]. The IES-R enables the measurement of 3 subscales: intrusion, avoidance, and hyperarousal. The reliability and validity of the Japanese version have been confirmed [25]. Cronbach's alpha reliability for this sample was 0.859 (total score). The cutoff point in the Japanese version is set at 24/25, and a total score equal to or above the cutoff point suggests posttraumatic stress disorder (PTSD).

#### 2.5.3. Profile of Mood States (POMS)

The Profile of Mood States (POMS) is a self-assessment questionnaire composed of 65 items designed to evaluate temporary emotional states. The questionnaire was developed by McNair [26] and enables the assessment of emotional state using 6 scales: tension-anxiety, depression-depressed mood, anger-hostility, vigor, fatigue, and confusion. The reliability and validity of the Japanese version have been confirmed [27]. Cronbach's alpha reliability for this sample was 0.775 (total score). The frequency of the mood corresponding to each item during the past week is rated on a five-point scale that ranges from “never (score 0)” to “very often (score 4).” The scores of all the items for each scale are totaled [28]. A higher total score indicates a higher intensity of mood in that category.

### 2.6. Statistical Analysis

#### 2.6.1. Comparison between the Intervention and Control Groups at Baseline

To compare variables and the scores for each scale at baseline between the two groups, the normality test and either the *χ* ^2^ test, *t*-test, or Mann-Whitney *U*-test were used.

#### 2.6.2. Evaluation of Responses to Group Intervention Approach

Intergroup differences in the score for each scale immediately before, immediately after, and 3 months after the intervention were analyzed using a two-way analysis of variance (analysis of nonpaired factors and paired factors) with the magnitude of the change in each scale score [(score immediately after intervention-baseline score) and (score 3 months after intervention-baseline score)] serving as a dependent variable.

All the *P* values were two tailed, and *P* values <.05 were considered significant. The Statistical Package for the Social Sciences (SPSS) software ver. 17.0J for Windows was used to perform all the statistical analyses.

### 2.7. Ethical Considerations

The protocol for this study was submitted to the nursing director of each of the five participating hospitals and was approved by the ethics committee of each hospital prior to the start of the study. Each candidate nurse was informed about the study using a leaflet stating the objectives and methods of the study, the design of the intervention, the capability and right of each nurse to refuse participation at any time, the strict protection of privacy, the lack of any disadvantage to nurses refusing to participate, the capability and right of the nurse to revoke their consent to participate in the study at any time.

## 3. Results

### 3.1. Enrollment in the Study

During the survey period, participants were recruited from among nurses working at five facilities. Sixty-two nurses who satisfied the inclusion criteria and provided their informed consent were randomly allocated to either the intervention group (*n* = 30) or the control group (*n* = 32).

Five subjects from the intervention group were unable to remain in the study until the end, and the collection of the questionnaire at 3 months after intervention was not possible for seven subjects in the control group. Thus, a final evaluation was possible for 25 subjects in the intervention group and 25 subjects in the control group.

### 3.2. Examples of Violent Speech/Violence Identified during Discussions with the Group Intervention Approach

The episodes of violent speech/violence experienced by nurses were summarized as follows.

1. Physical violence:
  - a nurse was beaten by a dissatisfied patient using a bar or similar object,
  - a patient, whose demand was not satisfied, kicked the door of the nurses' station, grabbed a nurse by the collar, and used violence,
  - a patient suddenly slapped the face of a nurse when the nurse was engaged in the care of another patient,
  - a patient with nocturnal delirium beat a nurse,
  - a patient in a borderline case threw a chair at the nurse when care was delayed.
2. Violent speech:
  - a patient suddenly said “Die” or “Go away” in a loud voice.

The subjects exposed to these episodes judged them as being violent speech/violence if they exceeded certain levels, for example, “It went beyond the limit,” “I cannot deal with this patient any further,” “I am sure the patient is behaving this way intentionally,” or “This behavior is not acceptable.” Some nurses stated that when they reported the event to their superior, their superior answered “That happened because you treated the patient poorly” or “Your way of dealing with the patient is problematic.” These nurses continued to experience fear or self-loathing after exposure to violent speech/violence or felt regret and intense unhappiness, depending on the attitude of their superior.

### 3.3. Comparison between the Intervention and Control Groups at Baseline

A comparison of the variables and scores for each scale at baseline between the two groups revealed no significant intergroup differences in any of the variables or scores (Table 1).

### 3.4. Comparison of Changes in IES-R Scores between the Two Groups

Table 2 compares the changes in the IES-R scores (intrusion, avoidance, hyperarousal and total scores) during the period from immediately after until 3 months after the end of intervention between the intervention and control groups.

In a two-way analysis of variance, significant intergroup differences in the changes in scores were noted for both the interaction and main effects on the avoidance, hyperarousal, and total score scales and for the main effect on the intrusion scale.

### 3.5. Comparison of Changes in POMS Scores between the Two Groups

Table 3 compares the changes in the POMS scores (tension-anxiety, depression, anger, vigor, fatigue, confusion, and TMD scores) during the period from immediately after until 3 months after the end of intervention between the intervention and control groups.

In a two-way analysis of variance, significant intergroup differences in the changes in scores were noted for both the interaction and main effects on the tension-anxiety scale and for the main effect on the depression scale.

## 4. Discussion

### 4.1. Violent Speech/Violence Experienced by Nurses

The present results demonstrate that the nurses in the present study frequently endured violent speech/violence with no active countermeasures, endorsing the previous findings that nurses are at a high risk of exposure to violent speech/violence from patients. The present study additionally revealed that some changes occurred in the way the nurses dealt with patients or in their feelings toward the patients after being exposed to violent speech, saying “I avoided contact with the patient as much as possible,” “I was distressed just to see the face of the patient,” “I minimized talking with the patient,” and “I avoided contact with the patient, asking other staff members to perform my role as much as possible.” Thus, the events resulted in the nurses having negative stances toward services or actions related to patient care.

### 4.2. Efficacy of Intervention

When the changes in the IES-R scores following intervention were analyzed in this randomized controlled trial, significant intergroup differences were noted for intrusion, hyperarousal, avoidance, and total score. Intrusion and hyperarousal, as evaluated using the IES-R scales, refer to symptoms characterized by extreme cautiousness and timidity, an inability to sleep because of concern over the event, and the repeated recollection of the event during daily life. During the intervention, various measures for dealing with violent speech/violence were discussed, and participants talked freely in a friendly atmosphere about how to control posttraumatic events, emotions, and stress by themselves and obtained knowledge regarding posttraumatic events and the resulting stress. Through these steps, the participants gained confidence in their capability to face situations or events involving violent speech/violence appropriately in the future. Furthermore, by learning relaxation techniques, the symptoms that the participants had been suffering from seemed to be alleviated. 

In the analysis of the changes in the POMS scores after intervention, significant intergroup differences were noted for anxiety and depression. The POMS system is designed to evaluate emotional status at a given time. Anxiety about possible violent speech/violence in the future and depression resulting from such anxiety seems to have been alleviated through frank discussions regarding the affliction and associated anxiety, learning about violent speech/violence, exchanging information on ways to overcome such events, and learning relaxation techniques in a group of nurses with similar experiences organized under a group intervention approach. The efficacy of the group intervention approach for group psychotherapy, which focuses on changing emotions, has been demonstrated in a previous study [17, 29, 30]. This approach seems to have yielded a similar efficacy among the nurses exposed to violent speech/violence in the present study.

### 4.3. Limitations of This Study

This study has several limitations. First, the analysis of differences in the characteristics, safety management system, safety practices, and other factors among the participating facilities was inadequate. Second, we cannot rule out the possibility that the subjects did not have a full understanding of the definition of violent speech/physical violence, though we endeavored to provide a very concrete definition and explanation. Third, a double-blind design could not be adopted for this study because of its nature, possibly resulting in a lack of adequate care or attention when arranging and implementing the study. That is, the authors assumed all the roles performed in the study, ranging from inviting the nurses to participate in the study to the allocation of the subjects to the intervention and control groups as well as the implementation of the intervention and evaluation. Thus, the reliability of the findings can be argued. In addition, all the study subjects were nurses working in psychiatric departments. The psychological problems arising from violent speech/violence experienced by nurses working in other specialties should also be investigated and overcome in future studies.

## 5. Conclusions

The present results suggest that the group intervention approach is an effective means of alleviating the psychological impact and stress of nurses exposed to violent speech/physical violence caused by patients. This approach can contribute to the improvement of the mental health of nurses, thereby improving the quality of nursing care provided to patients.

## Figures and Tables

**Table 1:** Comparison between baseline data in the intervention group and the control group.

Variables		Intervention group	Control group	*P* ^ (a)^
		(*n* = 30)	(*n* = 32)	
Gender				
Male		11	12	.95
Female		19	20	
Spouse				
Presence		17	18	.98
Absence		13	14	

		Median (range)		*P* ^ (b)^

Age (y)		33.0 (20–59)	29.0 (19–59)	.08
Length of nursing experience (months)		85.5 (14–435)	72.0 (13–469)	.94
Length of work in the psychiatry department (months)		47.5 (14–346)	54.5 ( 8–353)	.78
Interval between the time of exposure to the violent speech/violence and the present (months)		5.0 (1–180)	6.0 (1–36)	.64
Number of persons in the household		3.0 (1–8)	2.5 (1–6)	.18
Number of persons who provided social support		2 (1–4)	2 (1–4)	.26
Degree of satisfaction with support by family		2 (1–4)	2 (1–4)	.31
Degree of satisfaction with support by acquaintances		2 (1–4)	2 (1–4)	.70

		Mean (standard deviation)		*P* ^ (c)^

IES-R^(d)^				
Intrusion		4.77 (4.28)	6.62 (3.85)	.18
Avoidance		5.50 (4.13)	6.84 (4.04)	.20
Hyperactivity		5.17 (3.28)	5.43 (3.20)	.74
Total		15.43 (1.97)	18.44 (1.69)	.25
POMS^(e)^				
Tension-Anxiety		8.30 (2.70)	8.90 (4.21)	.51
Depression		15.93 (11.21)	14.91 (7.77)	.68
Anger		12.50 (10.23)	13.00 (7.03)	.83
Vigor		12.90 (7.35)	13.78 (6.17)	.61
Fatigue		13.42 (7.72)	13.13 (5.99)	.86
Confusion		11.70 (5.18)	12.09 (4.81)	.76
Total		56.80 (37.62)	58.00 (26.78)	.89

^(a)^ *χ* ^2^ test.

^(b)^Mann-Whitney *U*-test.

^(c)^ *t*-test.

^(d)^Impact of Event Scale—Revised.

^(e)^Profile of Mood States.

**Table 2:** Changes in the IES-R scores from immediately after completion of the intervention to 3 month after completion of the intervention.

	Time		Effects					
	Score change^(a)^	Score change^(b)^	Interaction			Main effect		
	(Immediately after intervention)	(1 month after intervention)	Group × time			Group		
	Mean (SD)	Mean (SD)	Freedom	*F* ^ (c)^	*P*	Freedom	*F* ^ (c)^	*P*

*Intrusion*								
Intervention group	−1.47 (2.46)	−1.70 (2.07)	2	2.90	.058	1	4.28	.040
Control group	−0.53 (1.97)	−0.38 (2.81)						
*Avoidance*								
Intervention group	−2.10 (2.71)	−2.03 (2.70)	1.58^(d)^	6.30	.005	1	7.96	.006
Control group	−0.22 (1.67)	−0.66 (2.40)						
*Hyperactivity*								
Intervention group	−1.50 (1.73)	−2.10 (2.04)	2	8.64	<.001	1	11.55	.001
Control group	−0.59 (1.97)	0.63 (2.40)						
*Total*								
Intervention group	−5.07 (4.91)	−5.83 (5.21)	2	10.00	<.001	1	15.49	<.001
Control group	−1.34 (3.65)	−0.97 (5.00)						

^(a)^[Score immediately after intervention]−[Baseline score].

^(b)^[Score 3 months after the completion of the intervention]−[Baseline score].

^(c)^ *F* statistic in repeated measures analysis of variance.

^(d)^Greenhouse-Geisser correction.

**Table 3:** Changes in the POMS scores from immediately after completion of the intervention to 3 month after completion of the intervention.

	Time		Effects					
	Score change^(a)^	Score change^(b)^	Interaction			Main effect		
	(Immediately after intervention)	(1 month after intervention)	Group × time			Group		
	Mean (SD)	Mean (SD)	Freedom	*F* ^ (c)^	*P*	Freedom	*F* ^ (c)^	*P*

*Tension-anxiety*								
Intervention group	−1.87 (4.95)	−2.97 (4.16)	2	30.01	<.001	1	53.55	<.001
Control group	5.47 (5.23)	6.62 (6.10)						
*Depression*								
Intervention group	−0.07 (8.17)	−0.73 (8.19)	2	2.36	.098	1	4.64	.035
Control group	3.88 (5.23)	3.59 (9.36)						
*Anger*								
Intervention group	−2.43 (8.80)	−2.10 (9.92)	1.35^(d)^	0.85	.845	1	0.02	.881
Control group	−2.40 (11.01)	−2.93 (10.27)						
*Vigor*			2	1.50	.227	1	2.49	.120
Intervention group	−0.17 (3.94)	−1.37 (6.66)						
Control group	1.09 (8.05)	0.50 (5.98)						
*Fatigue*			2	0.86	.426	1	1.14	.288
Intervention group	−0.60 (4.43)	−1.77 (6.21)						
Control group	−2.53 (6.90)	−2.75 (6.05)						
*Confusion*			2	0.62	.537	1	1.14	.289
Intervention group	−0.47 (3.44)	−1.07 (4.33)						
Control group	−1.40 (5.42)	−2.34 (5.17)						
*Total*			1.76^(d)^	0.75	.452	1	0.26	.608
Intervention group	−2.57 (21.00)	−3.70 (30.45)						
Control group	−2.71 (32.20)	3.31 (27.60)						

^(a)^[Score immediately after intervention]−[Baseline score].

^(b)^[Score 3 months after the completion of the intervention]−[Baseline score].

^(c)^ *F* statistic in repeated measures analysis of variance.

^(d)^Greenhouse-Geisser correction.
